# Body piercing and adolescent risk-taking: association or expression?

**DOI:** 10.1007/s00431-026-07115-x

**Published:** 2026-06-03

**Authors:** Meltem Ileri, Hüsniye Yücel, Deniz Yücel, Rola Tokan, Ayşegül Şahiner, Gülsüm Ozen, Semra Cetinkaya, Ayşe Gül Güven, Zehra Aycan

**Affiliations:** 1 Department of Pediatrics, Ankara Etlik City Hospital, Ankara, Ankara Turkey; 2 Department of Adolescent Health, Ankara University Institute of Health Sciences, Ankara, Ankara Turkey; 3 Medical School, School of Medicine, University of Köln, Köln, Germany; 4 Department of Pediatrics, Beştepe Public Hospital, Ankara, Turkey; 5 Department of Pediatrics, Yenimahalle Research and Education Hospital, Ankara, Turkey; 6 Department of Pediatrics, Atatürk Sanatoryum Research and Education Hospital, Ankara, Turkey; 7 Department of Adolescent Health, Ankara University School of Medicine, Ankara, Turkey

**Keywords:** Children, Risky behavior, Adolescent risk taking, Piercings, Suicide risk

## Abstract

This study aimed to investigate the relationship between body piercing and risk-taking behaviors among adolescents, while assessing their knowledge and attitudes regarding piercing practices, hygiene, and potential complications. This cross-sectional descriptive study was conducted among 141 adolescents aged 12–19 years attending an adolescent health clinic between February and November 2025. Participants were divided into two groups: adolescents with at least one body piercing (*n* = 71) and controls without piercings (*n* = 70). Data on sociodemographic characteristics, risk-taking behaviors, and mental health status were collected using a structured questionnaire. Risk behaviors were defined as meeting in person with someone first met online, driving without a seat belt, getting into a stranger’s vehicle, experimenting with cigarettes, alcohol, or substances, and having suicidal thoughts. Suicide risk was evaluated using the “Ask Suicide-Screening Questions.” Knowledge regarding piercing procedures, hygiene, and complications was assessed through an 18-item survey, and scores were calculated on a 100-point scale. Adolescents with piercings demonstrated significantly higher engagement in risk-taking behaviors compared to controls, including smoking (73.2% vs. 11.4%), alcohol use (63.4% vs. 8.6%), substance use (11.3% vs. 0%), and non-suicidal self-injury (42.3% vs. 4.3%) (all *p* < 0.001). Positive suicide risk screening was also more frequent in the piercing group (45.1% vs. 7.1%, *p* < 0.001). Nearly half of the pierced adolescents reported local complications such as hyperemia, swelling, or pain, and only 49.3% had their procedures performed in licensed studios. The overall knowledge score was moderate (mean = 59.4 ± 14.2), with the lowest accuracy in the “Site and Setting of Application” domain. None of the participants answered all questionnaire items correctly.

*Conclusion*: Although body piercing has become a normalized form of self-expression among adolescents, it remains associated with significantly higher rates of risk-taking behaviors and psychological vulnerability. Moreover, adolescents demonstrated insufficient knowledge about safe piercing practices, sterilization, and complications, and most had not received information from health professionals. These findings highlight the need for targeted educational interventions and counseling to promote safer body modification practices and to use piercing as an opportunity for psychosocial assessment in adolescent health care.

**Table:** 

**What is Known:** • *Body piercing is a common form of self-expression among adolescents and has been previously linked to certain risk taking behaviors like smoking and substance use. * • *Adolescents generally demonstrate insufficient knowledge regarding hygiene, sterilization, and health complications associated with body modifications.*
**What is New:** • *This study explicitly details a alarmingly high rate of positive suicide risk screening and non-suicidal self-injury specifically among pierced adolescents.* •*It quantifies a distinct gap in knowledge regarding the Site and Setting of Application, revealing that more than half of adolescents utilize unlicensed studios without professional health guidance.* •*Body piercing should serve as a practical, visible trigger for pediatricians to initiate comprehensive psychosocial and mental health screenings in clinical settings.*

**Supplementary Information:**

The online version contains supplementary material available at 10.1007/s00431-026-07115-x.

## Introduction

Body modification practices such as tattooing and body piercing have become increasingly common forms of self-expression among adolescents and young adults worldwide. While in some contexts these practices have been perceived as markers of rebellion or deviant subcultures, they are increasingly understood as socially meaningful expressions of identity, autonomy, and esthetic preference [1]. Despite this normalization, body piercing among adolescents continues to attract clinical and psychosocial attention due to its potential health risks and its debated link to risk-taking or sensation-seeking behaviors [2, 3]. Earlier epidemiological studies associated tattoos and piercings with increased likelihood of alcohol or drug use, unsafe sexual activity, violence, and other risky acts [4]. However, recent evidence indicates that the previously observed associations between body modification practices and deviant or oppositional identities are diminishing, as these practices have become increasingly normalized and integrated into mainstream culture, reflecting esthetic or personal expression rather than social nonconformity [5]. This shift necessitates a comprehensive investigation into the multifaceted nature of body piercing, considering both its association with risk behaviors and its role as a medium for identity and individuality during adolescence.

Historically, body piercing is a practice deeply rooted in antiquity, with archaeological evidence of various body modifications across diverse cultures and millennia. These procedures have served as powerful symbols of tribal status, religious identity, beauty, and rites of passage, such as the transition into adulthood [5]. While these practices have been integral to cultural and magical rituals in many societies for thousands of years, much of the modern clinical and psychosocial research focusing on the relationship between adolescent body piercing and risk-taking behaviors stems primarily from high-income Western countries [2]. This geographic concentration in literature leaves a significant gap in understanding these phenomena within non-Western contexts, where different cultural traditions and religious beliefs may influence the perception and prevalence of body art [6, 7].

This study significantly advances the existing literature by integrating recent empirical findings on the complex association between body art and risk-taking behaviors [8, 9]. Unlike previous research that predominantly focuses on high-income Western populations, our work explicitly addresses critical knowledge gaps within non-Western contexts by incorporating data from a Turkish setting.

Although the popularity of body piercing and tattooing has increased substantially among young people, several studies have demonstrated that adolescents often have insufficient knowledge about the potential health risks and hygiene requirements of these practices, and they tend to rely on non-professional or unreliable sources of information rather than evidence-based guidance from health authorities or professionals [10, 11].

The study aimed to compare risk behaviors among adolescents with and without piercings, as well as to explore motivations for obtaining piercings and to assess level of knowledge regarding piercing-related health risks and procedures.

## Materials and methods

### Study design

This cross-sectional descriptive study was conducted at the Adolescent Health Outpatient Clinic of a tertiary university hospital located in Ankara, Turkey between February and November 2025. The study included adolescents aged 12 to 19 years who presented to the clinic during the study period. Participants were divided into two groups: adolescents with at least one body piercing (*n* = 71) and controls without any piercing (*n* = 70). Body piercing is defined as a form of body modification involving the intentional perforation of the skin, underlying adipose tissue, or cartilage to facilitate the insertion of jewelry or decorative ornaments. Ear piercing is the process of perforating the auricular cartilage for decorative purposes using sterile needles or modern devices, followed by the insertion of jewelry into the resulting opening.

Participants were selected from adolescents attending the adolescent outpatient clinic and pediatric inpatient unit for any medical reason. The inclusion criteria for the study group consisted of having at least one body piercing and providing informed consent to participate. The control group was composed of adolescents attending the same clinic who did not have any piercings and agreed to participate in the study. No specific medical condition was used as an exclusion criterion unless it prevented the participant from providing reliable survey responses.

### Outcome measures

Sociodemographic characteristics (age, sex, educational status, and family structure), presence of chronic illness, and self-reported risk-taking behaviors were recorded for all participants. In the piercing group, detailed information was collected regarding the characteristics of piercing practices, post-procedure complications, and individual motivations for undergoing piercing. The questionnaire regarding risk-taking behaviors was developed by researchers based on current literature, and the complete set of questions has been included in the Supplementary Materials section. Rather than referring to a specific timeframe, the assessment of risk-taking behaviors pertained to any point in the participant’s lifetime. In this study, risk-taking behaviors were defined as meeting in person with someone first encountered online, driving without wearing a seat belt, getting into a stranger’s vehicle, experimenting with cigarettes, alcohol, or other substances, having non-suicidal self-injury (NSSI) and having suicidal thoughts. NSSI refers to the intentional destruction of one’s own body tissue without suicidal intent and for purposes not socially approved [12]. Suicidal ideation was evaluated using the Ask Suicide-Screening Questions (ASQ) toolkit, a validated instrument designed to identify individuals at risk.

Suicide risk was assessed using the ASQ tool developed by the National Institute of Mental Health [13]. The ASQ is a brief four-item instrument validated for use in adolescents, with any affirmative response indicating the need for a comprehensive suicide risk evaluation [14]. Adolescents who screened positive on the ASQ (i.e., answered “yes” to any of the four screening questions) underwent further evaluation by a qualified mental health professional. The data collection instrument utilized in this study was previously validated in the language of data collection ensuring their reliability and cultural appropriateness for the target population [14]. The secondary assessment included a comprehensive suicide risk evaluation and a brief psychosocial assessment focusing on depression, anxiety, substance use, and family or peer stressors. While the ASQ was used to screen for presence of suicide risk as a binary outcome variable, those who screened positive received further evaluation as a critical ethical and safety function, ensuring that any adolescent identified as high-risk through the ASQ received immediate clinical attention and appropriate psychiatric referral in accordance with institutional safety protocols.

Participants’ knowledge about site and setting of piercing application; hygiene and sterilization practices during piercing; and health risks and complications associated with piercing was assessed using a structured questionnaire consisting of 18 closed-ended questions. The application score was calculated on a 100-point scale using the formula (i.e., (*Q*1 + *Q*2 + *Q*3)/*3* × *100*). The total score was obtained by summing the responses to all 18 items, dividing by the total number of items, and multiplying the result by 100. The assessment instrument utilized in this study was a structured “Knowledge, Attitude, and Practice questionnaire” specifically developed by the researchers based on a comprehensive review of the current literature [10, 11]. To ensure the reliability of the reported findings, the knowledge levels were quantified on a 100-point scale. The categorization of knowledge levels was defined as follows: *Low Knowledge:* Scores falling below 50% of the total points; *Moderate Knowledge: S*cores ranging between 50 and 75%, representing an intermediate understanding of hygiene and health risks; *High Knowledge:* Scores exceeding 75%, indicating a comprehensive grasp of sterilization and complication management. This systematic scoring method facilitates a standardized qualitative analysis, allowing for a precise interpretation of the participants' awareness regarding piercing-related complications.

Comparisons were made between the piercing and control groups to evaluate differences in sociodemographic variables and risk-taking behaviors.

### Statistical analysis

Statistical analyses were conducted using SPSS version 25.0 (IBM Corp., Armonk, NY, USA). Continuous variables were presented as mean and standard deviation, whereas categorical variables were expressed as frequencies and percentages. The normality of the data distribution was assessed using the Shapiro–Wilk test. Categorical variables were compared using the Chi-square test, whereas continuous variables between two independent groups were analyzed using the independent samples *t*-test. The Adolescent Risk-Taking Questionnaire (ARQ) was administered to all participants in the study group. “Knowledge, Attitude, and Practice questionnaire” was administered to the participants in the piercing group. Differences among the three dimensions of the “Knowledge, Attitude, and Practice questionnaire” (Application, Hygiene, and Complications), measured within the same individuals, were analyzed using repeated measures one-way ANOVA, followed by Bonferroni-adjusted post-hoc comparisons. The internal consistency of the 18-item dichotomous knowledge and attitudes test was evaluated using the KR-20 coefficient. KR-20 values were 0.73 for “Applications” dimension, 0.92 for “Hygiene” dimension, and 0.91 for “Complications” dimension. The overall KR-20 coefficient for the full test was 0.95, indicating high internal reliability. Internal consistency of this scale was assessed using Cronbach’s alpha. The “Knowledge, Attitude, and Practice questionnaire” demonstrated good reliability in the current sample (*α* = 0.83).

Multivariable logistic regression analyses were performed to examine the association between piercing status and various risk behaviors. Each risk behavior (smoking, alcohol use, internet-based face-to-face interaction, non-suicidal self-injury [NSSI], ASQ positivity, and seatbelt use) was analyzed as a separate dependent variable. Piercing status was included as the main independent variable in all models. Age was entered as a continuous variable, while gender and parental marital status were included as categorical variables using appropriate reference categories. Odds ratios (ORs) with 95% confidence intervals (CIs) and corresponding *p*-values were reported. All models were adjusted for age, gender, and parental marital status. A *p*-value of < 0.05 was considered statistically significant.

Regarding the assessment of motivations for undergoing body piercing, the data collection instrument utilized a multiple-response format, allowing participants to select more than one option. The responses were subsequently categorized based on specific thematic piercing motivations. Motivations were categorized into distinct thematic domains (e.g., esthetic, psychological, or socio-cultural) based on established conceptual frameworks. In the statistical analysis, the highest frequency rates were employed to identify and prioritize the primary reasons cited by the participants.

To account for potential confounding variables, a multivariable logistic regression was performed to evaluate the association between piercing status and risk behaviors. In this model, “piercing status” was treated as the primary predictor, while “risk behaviors” served as the dependent variable.

### Ethical approval

Ethical approval for the study was obtained from the institutional ethics committee; the Ankara University Health Sciences Scientific Research and Publication Ethics Committee (Meeting date: February 17, 2025; Meeting No: 04; Decision No: 82) prior to data collection. Written informed consent was obtained from all participants and their parents or legal guardians, in accordance with the Declaration of Helsinki. No funding was secured for this study.

## Results

Total of 141 adolescents were included in the study: 71 in the piercing group and 70 in the control group. The mean age of the participants was significantly higher in the piercing group compared with the control group (17.0 ± 1.2 vs. 15.0 ± 1.5 years, *p* < 0.001). The majority of adolescents with piercings were female (81.7%), whereas males were more frequent in the control group (34.3%, *p* = 0.031). Adolescents with piercings were more likely to have discontinued school (26.8% vs. 1.4%, *p* < 0.001) and to be employed in a job (20.0% vs. 1.4%, *p* = 0.004). Chronic illness was more common among the piercing group (31.0% vs. 15.7%, *p* = 0.032), and parental divorce or widowhood was significantly higher in this group (52.1% vs. 8.6%, *p* < 0.001).

Maternal history of tattoos (19.7% vs. 2.9%, *p* = 0.002) and piercings (14.1% vs. 4.3%, *p* = 0.044) was more prevalent among adolescents with piercings, whereas paternal body modification did not differ significantly between groups (Table 1).

**Table 1:** Demographic characteristics, habits, and risky behaviors of the study group

Variable	Piercing group (*n* = 71) *n* (%)	Control group (*n* = 70) *n* (%)	*P*
**Gender**			
Male	13 (18.3)	24 (34.3)	0.031
Female	58 (81.7)	46 (65.7)	
**Age group**			
12–15 years-old	6 (8.5)	48 (68.6)	** < 0.001**
16–19 years-old	65 (91.5)	22 (31.4)	
**Mean age (years old)**	17.0 ± 1.2	15.0 ± 1.5	** < 0.001**
**School attendance**			
Yes	52 (73.2)	69(98.6)	** < 0.001**
No	19 (26.8)	1 (1.4)	
**Working in a job**			
Yes	5 (20.0)	1 (1.4)	**0.004**
No	20 (80.0)	69 (98.6)	
**Having chronic disease**	22 (31.0)	11 (15.7)	**0.032**
**Parental marital status**			
Married	34 (47.9)	64 (91.4)	** < 0.001**
Divorced or widowed	37 (52.1)	6 (8.6)	
**Mothers having**			
**Tattoos**	14 (19.7)	2 (2.9)	**0.002**
**Piercings**	10 (14.1)	3 (4.3)	**0.044**
**Fathers having**			
**Tattoos**	9 (12.7)	6 (8.6)	**0.420**
**Piercings**	1 (1.4)	1 (1.4)	**0.999**
**Habits and risky behaviors**			
Tried smoking	52 (73.2)	8 (11.4)	** < 0.001**
Regular smoking	38 (53.5)	3 (4.3)	** < 0.001**
Alcohol use	45 (63.4)	6 (8.6)	** < 0.001**
Substance use	8 (11.3)	0 (0)	**0.006**
Travel in car without seatbelt	38 (53.5)	29 (41.4)	**0.151**
Face-to-face meeting someone met online	37 (52.1)	6 (8.6)	** < 0.001**
NSSI	30 (42.3)	3 (4.3)	** < 0.001**
ASQ Positive screening	32 (45.1)	5 (7.1)	** < 0.001**

*NSSI* Non-suicidal self-injury, *ASQ* Ask Suicide-Screening Questions

### Risky behaviors and mental health screening

Adolescents with piercings reported substantially higher engagement in risk behaviors compared to controls (Table 1). The prevalence of ever having smoked (73.2% vs. 11.4%, *p* < 0.001), regular smoking (53.5% vs. 4.3%, *p* < 0.001), alcohol use (63.4% vs. 8.6%, *p* < 0.001), and substance use (11.3% vs. 0%, *p* = 0.006) were all markedly greater among the piercing group. Similarly, meeting people in person who were first met online (52.1% vs. 8.6%, *p* < 0.001) and engaging in non-suicidal self-injury (NSSI) behaviors (42.3% vs. 4.3%, *p* < 0.001) were significantly more frequent.

Positive screening results on the ASQ were observed in 45.1% of adolescents with piercings, compared to only 7.1% of controls (*p* < 0.001), indicating a higher risk for suicidal ideation or behavior in this group. No significant difference was found between groups regarding traveling in a car without a seatbelt (*p* = 0.151).

Multivariable logistic regression analyses were conducted to examine the association between piercing status and various risk behaviors, adjusting for age, gender, and parental marital status (Table 2).

**Table 2:** Multivariable logistic regression analyses examining the association between piercing status and risk behaviors

Variables	Smoking status	Alcohol use	Meeting person met online	NSSI	ASQ	Seatbelt use
	OR (95% CI)	OR (95% CI)	OR (95% CI)	OR (95% CI)	OR (95% CI)	OR (95% CI)
	*p*-value	*p-*value	*p-*value	*p-*value	*p*-value	*p-*value
Piercing (group)	8.40 (2.70–26.13) < 0.001	10.94 (3.19–37.49) < 0.001	5.20 (1.63–16.61) 0.005	12.60 (2.70–58.83) 0.001	7.53 (2.04–27.78) 0.002	1.46 (0.58–3.70) 0.426
Age (years)	1.58 (1.11–2.25) 0.010	1.51 (1.07–2.14) 0.019	1.33 (0.96–1.86) 0.087	0.95 (0.66–1.37) 0.792	0.99 (0.70–1.39) 0.943	0.96 (0.74–1.23) 0.724
Gender (female vs male)	0.45 (0.14–1.44) 0.179	0.33 (0.10–1.04) 0.059	0.78 (0.28–2.18) 0.637	1.77 (0.55–5.70) 0.337	1.26 (0.44–3.62) 0.668	1.01 (0.47–2.19) 0.982
Parental marital status (divorced/widowed vs married)	3.49 (1.28–9.49) 0.014	1.52 (0.60–3.84) 0.378	2.38 (0.99–5.77) 0.054	2.04 (0.80–5.20) 0.134	2.28 (0.93–5.62) 0.072	1.61 (0.71–3.66) 0.256

*OR* odds ratio, *CI* confidence interval, *NSSI* non-suicidal self-injury, *ASQ* Ask Suicide-Screening Questions

All models were adjusted for age (continuous), gender, and parental marital status

Reference categories: male for gender; married for parental marital status; control group for piercing status

Piercing status was found to be significantly associated with several risk behaviors. Individuals in the piercing group had significantly higher odds of smoking (OR = 8.40, 95% CI 2.70–26.13, *p* < 0.001), alcohol use (OR = 10.94, 95% CI 3.19–37.49, *p* < 0.001), meeting someone met online (OR = 5.20, 95% CI 1.63–16.61, *p* = 0.005), NSSI (OR = 12.60, 95% CI 2.70–58.83, *p* = 0.001), and ASQ positivity (OR = 7.53, 95% CI 2.04–27.78, *p* = 0.002). No statistically significant association was observed between piercing status and seatbelt use (OR = 1.46, 95% CI 0.58–3.70, *p* = 0.426).

Age was significantly associated with smoking (OR = 1.58, 95% CI 1.11–2.25, *p* = 0.010) and alcohol use (OR = 1.51, 95% CI 1.07–2.14, *p* = 0.019), indicating increased odds of these behaviors with increasing age. However, age was not significantly associated with other outcomes.

Gender was not significantly associated with any of the examined risk behaviors, although a borderline association was observed for alcohol use (*p* = 0.059).

Parental marital status was significantly associated with smoking (OR = 3.49, 95% CI 1.28–9.49, *p* = 0.014), indicating higher odds of smoking among individuals with divorced or widowed parents. No statistically significant associations were observed between parental marital status and other outcomes, although borderline associations were noted for internet-related behaviors (*p* = 0.054) and ASQ positivity (*p* = 0.072).

Overall, piercing status remained a strong and independent factor associated with multiple risk behaviors after adjusting for sociodemographic variables.

### Characteristics of piercing practices

Among adolescents with piercings, 32.4% had four or more piercings, and another 32.4% had only one. The first piercing was typically obtained between ages 12–15 years (70.4%). One-third (32.4%) of the adolescents had both tattoos and piercings. The most common piercing sites were the nose (45.1%) and ears (38.0%), followed by the lip (15.5%), umbilicus (9.9%), and eyebrow (9.9%).

Nearly half of the adolescents experienced local complications following the procedure, most commonly hyperemia (49.3%), swelling (45.1%), and pain (32.4%). Less frequent adverse outcomes included discharge (14.1%), bruising (12.7%), and embedding (4.2%) (Table 3).

**Table 3:** Body piercing practices and safety precautions

Number of piercings	*n*	%
1	23	**32.4**
2	14	**19.7**
3	11	**15.5**
≥ 4	23	**32.4**
**First piercing age (years old)**		
≤ 11	6	**8.5**
12–15	50	**70.4**
16–19	15	**21.1**
**Having both tattoos and piercing**	23	**32.4**
**Piercing sites**		
• Nose	32	**45.1**
• Ear	27	**38.0**
• Lip	11	**15.5**
• Umbilicus	7	**9.9**
• Eyebrow	7	**9.9**
• Oral cavity	5	**7.0**
• Tongue	4	**5.6**
**Complications**		
• Hyperemia	35	**49.3**
• Swelling	32	**45.1**
• Pain	23	**32.4**
• Localized warmth	14	**19.7**
• Discharge	10	**14.1**
• Bruising	9	**12.7**
• Color change	4	**5.6**
• Embedding	3	**4.2**
• Fever	1	**1.4**
**Perceived meanings of piercing**		
• Physical expression	28	**39.4**
• Fashion	28	**39.4**
• No meaning	21	**29.6**
• Self-expression	19	**26.8**
• Courage	14	**19.7**
• Being different	14	**19.7**
• Rebellion	12	**16.9**
• Independence	9	**12.7**
• Sexual	4	**5.6**
• Religious	3	**4.2**
• Peer influence	1	**1.4**
**Piercing studios**		
Officially licensed	35	**49.3**
Written parental consent	23	**32.4**
Verbal parental consent	36	**50.7**
Screening for diseases	24	**33.8**
Blood analysis before procedure	2	**2.8**
**Information about piercing**		
From pediatrician	4	**5.6**
From physician	7	**9.9**
From other sources	33	**46.5**

### Perceptions, motivations, and safety practices

The perceived meanings of piercing varied across participants, with “fashion” (39.4%) and “physical expression” (39.4%) being the most common reasons, followed by “no particular meaning” (29.6%) and “self-expression” (26.8%). Motivations such as courage (19.7%), being different (19.7%), and rebellion (16.9%) were also reported, while few adolescents cited sexual (5.6%) or religious (4.2%) reasons. Peer influence was rarely reported (1.4%).

Regarding safety and procedural standards, only 49.3% of piercings were performed in legally licensed studios. Written parental consent was obtained in 32.4% of cases, while verbal consent was more common (50.7%). Disease screening before piercing was reported by 33.8% of participants, and only 2.8% underwent pre-procedure blood analysis. Most adolescents obtained information about piercing from non-medical sources (46.5%), whereas only 5.6% were informed by pediatricians and 9.9% by physicians (Table 3).

### Knowledge and awareness

Knowledge assessment revealed substantial gaps in adolescents’ understanding of safe piercing practices. The results are given in Table 3. While some participants were aware of the importance of professional settings and sterilization, many lacked sufficient knowledge regarding infection risks and preventive measures. The mean knowledge score was moderate, reflecting limited awareness of potential health complications and safety requirements during piercing procedures (Table 4).

**Table 4:** Knowledge and attitudes of adolescents regarding body piercing practices and safety

Questions		True	False	Null	Score			Statistical test
		*n* (%)	*n* (%)	*n* (%)	Mean ± SD	Median	(min–max)	
Application	The piercing procedure should be performed in fixed locations such as jewelry shops or tattoo studios	13 (18.3)	54 (76.1)	4 (5.6)	32.4 ± 32.1	33.3	(0–100)	*F* = 67.756 *p* < 0.001 There is a statistically significant difference between all three groups
	The piercing procedure should be performed in a licensed institutions	23 (32.4)	42 (59.2)	6 (8.5)				
	The piercing procedure should be performed in private health institutions	32 (45.1)	35 (49.3)	4 (5.6)				
Hygiene	There is no need to pay attention to skin cleanliness during the piercing procedure	68 (95.8)		3 (4.2)	76.3 ± 21.3	80.0	(20–100)	
	It is sufficient for the jewelry used in piercing to be clean	53 (74.6)	16 (22.5)	2 (2.8)				
	After skin disinfection, piercing should be performed under sterile (germ-free) conditions and with sterile equipment	65 (91.5)	1 (1.4)	5 (7.0)				
	The instruments used during the piercing procedure should be clean, but they do not need to be sterile	56 (78.9)	12 (16.9)	3 (4.2)				
	Sterility procedures should vary depending on the area where the piercing is performed	25 (35.2)	38 (53.5)	8 (11.3)				
Complications	Infection is one of the possible complications associated with piercing	66 (93.0)	3 (4.2)	2 (2.8)	59.1 ± 17.4	60.0	(0–100)	
	Bleeding is not among the possible complications associated with piercing	60 (84.5)	8 (11.3)	3 (4.2)				
	Transmission of HIV/Hepatitis B–C is one of the possible complications associated with piercing	32 (45.1)	18 (25.4)	21 (29.6)				
	Tetanus is not among the possible complications associated with piercing	37 (52.1)	22 (31.0)	12 (16.9)				
	Allergic reactions and rejection of the jewelry by the body are among the possible complications associated with piercing	64 (90.1)	4 (5.6)	3 (4.2)				
	Tissue damage (such as tearing or tooth fracture) is not among the possible complications associated with piercing	54 (76.1)	11 (15.5)	6 (8.5)				
	Swallowing oral piercings is not among the possible complications associated with piercing	57 (80.3)	9 (12.7)	5 (7.0)				
	An unaesthetic or unsightly appearance due to improper technique is among the possible complications associated with piercing	13 (18.3)	34 (47.9)	24 (33.8)				
	After oral piercing, inflammation of the heart lining (endocarditis) may occur	16 (22.5)	45 (63.4)	10 (14.1)				
	After oral piercing, tongue swelling or airway obstruction does not occur	15 (21.1)	50 (70.4)	6 (8.5)				
Total score					59.4 ± 14.2	58.3	(5.6–88.9)	

Among adolescents, the highest level of correct knowledge was observed regarding hygiene and infection-related items. The vast majority correctly identified that piercing should be performed under sterile conditions following skin disinfection (91.5%) and that infection is a possible complication (93.0%). Similarly, awareness of allergic reactions and body rejection as potential complications was high (90.1%).

In contrast, misconceptions and lack of awareness were notable in several areas. Only about one-third of participants correctly recognized that piercings should be performed in public (32.4%) or private (45.1%) health institutions, while most erroneously believed that procedures could be performed in jewelry shops or tattoo studios (76.1%). Knowledge about sterility procedures varying by anatomical site was also limited, with only 35.2% answering correctly.

The most commonly incorrect responses were related to bleeding (84.5%) and tissue damage (76.1%) not considered possible complications, and to the misunderstanding that sterilization standards differ by piercing area (53.5% incorrect). Furthermore, some participants lacked knowledge about the potential for HIV or Hepatitis transmission (45.1% correct) and tetanus risk (52.1% correct).

Items with the highest number of “no response” (null) answers included statements related to esthetic complications (33.8%) and endocarditis following oral piercing (14.1%), indicating uncertainty or lack of awareness in these domains. The overall mean knowledge score was 59.4 ± 14.2 (range = 56.6–88.9), indicating that their overall knowledge score was moderate about the procedures and complications.

The *Site and Setting of Piercing Application* domain was the least accurately known area, whereas the *Hygiene and Sterilization Practices During Piercing* and *Health Risks and Complications Associated with Piercing* domains demonstrated a moderate level of knowledge. None of the items were answered correctly by all participants.

## Discussion

This study examined the relationship between body piercing and risk-taking behaviors among adolescents, highlighting that pierced adolescents exhibited significantly higher engagement in various health-risk behaviors, including smoking, alcohol and substance use, NSSI, and positive suicide risk screening in the study group. These findings suggest that, although body piercing has become a mainstream practice, it may still serve as a behavioral marker for a subset of adolescents who demonstrate increased psychosocial vulnerability or a tendency toward sensation seeking. Furthermore, adolescents who had undergone piercing demonstrated limited knowledge about piercing procedures and associated complications and reported not receiving information or guidance from health professionals.

While tattoos and piercings are more common and socially accepted than ever before, concerns persist regarding their potential association with adolescent risk-taking behaviors [15, 16]. Earlier research suggested that adolescents with body modifications were more likely to engage in substance use, violence, unsafe sexual activity, and suicidal behaviors [9, 17]. The strong associations observed between body piercing and smoking, alcohol consumption, and substance use are consistent with previous research linking body modification with risk-taking and impulsive personality traits [18]. Similarly, the significantly higher rates of ASQ-positive screening and NSSI behaviors in the piercing group align with earlier findings indicating that adolescents engaging in body modification may also exhibit higher levels of psychological distress, depressive symptoms, or suicidal ideation [19, 20]. Equally important is the distinction between voluntary body modification and NSSI. Although both involve alteration of the body’s surface, NSSI typically reflects impulsive or compulsive behavior associated with emotional distress, whereas piercing is a planned, often socially sanctioned act with symbolic meaning. Intent therefore represents the key differentiator between the two phenomena [21]. Recognizing this distinction is essential for clinicians to avoid pathologizing normative adolescent behavior while still identifying individuals who may require psychosocial support.

However, the directionality of this association remains unclear—piercing may either reflect an underlying predisposition to risk-taking or represent a coping mechanism or symbolic act of autonomy in response to emotional stress [17]. One of the most notable findings of this study was the significantly higher prevalence of risk-taking behaviors among adolescents with body piercings compared to their non-pierced peers. This suggests that body piercing may serve as a behavioral marker for broader risk-taking tendencies in adolescents in the study population. Consistent with previous research conducted in the same community, these findings suggest that the prevalence of risk-taking behaviors among adolescents remains largely unchanged [7].

The elevated rates of school dropout and parental divorce among adolescents with piercings further indicate a potential link between family structure, psychosocial stressors, and engagement in body modification [22]. These contextual factors may contribute to both the behavioral and emotional dimensions of adolescent risk-taking. Addressing these underlying determinants through integrated psychosocial support and family-based counseling could mitigate associated risks. The findings of this study corroborate the recent position paper by the European Academy of Pediatrics (EAP) which underscores the high prevalence of body modifications among adolescents and their significant correlation with risk-taking behaviors. Our data, demonstrating increased rates of substance use, smoking, and positive suicide risk screening among pierced adolescents, align with the EAP’s observation that body modification often serves as behavioral markers for “novelty seeking” and psychosocial vulnerability [3].

The present study examined the association between piercing status and a range of risk behaviors while adjusting for key sociodemographic variables, including age, gender, and parental marital status. The findings demonstrate that piercing status is independently associated with multiple risk behaviors, including smoking, alcohol use, internet-related social interactions, NSSI, and ASQ positivity. Importantly, these associations remained statistically significant after controlling for potential confounders. This suggests that the observed relationships are not solely attributable to differences in age, gender, or family structure, but rather indicate a robust and independent link between piercing and risk-related behaviors. These findings are consistent with previous literature suggesting that body modification practices may be associated with broader patterns of risk-taking behavior.

In our study group, age was positively associated with smoking and alcohol use, which aligns with established evidence indicating increased engagement in such behaviors with advancing age. In contrast, gender was not significantly associated with most outcomes, although a borderline association was observed for alcohol use. Parental marital status was significantly associated with smoking, and showed borderline associations with certain other outcomes, suggesting that family structure may play a partial role in shaping risk behaviors.

In the current study, although several associations were strong, some confidence intervals were relatively wide. This likely reflects the limited number of observations within certain subgroups and the imbalance between comparison groups. Therefore, these estimates should be interpreted with caution. However, the consistency in the direction and magnitude of the associations across multiple models strengthens the overall validity of the findings.

Importantly, the present results support recent evidence that the meaning and motivation behind piercing among adolescents have shifted from rebellion or deviance toward self-expression, fashion, and esthetic preference. Nearly 40% of participants cited “fashion” or “physical expression” as their primary reason for obtaining piercings, and only a minority attributed it to rebellion or peer influence [23]. This transformation mirrors the broader cultural normalization of body modification practices in contemporary youth, where such acts may serve as tools for identity formation rather than overt defiance [24]. However, despite this normalization, awareness regarding health risks and safe piercing practices remained limited.

Nevertheless, the clinical and public health implications of these findings are considerable. Nearly half of the adolescents reported local complications such as hyperemia, swelling, or pain, while only half had their procedures performed in legally licensed studios. Inadequate sterilization and limited awareness of infectious risks highlight persistent gaps in adolescent knowledge and procedural safety. Similar to recommendations by the American Academy of Pediatrics (AAP), these results underscore the importance of anticipatory guidance by pediatricians and adolescent health professionals, who should provide nonjudgmental education about safe piercing practices, potential medical complications, and informed decision-making [5]. Despite their relative safety when performed under hygienic conditions, piercings can result in medical complications such as infection, allergic reactions, keloid formation, or delayed wound healing. The AAP recommends that adolescents be educated about sterile techniques, qualified practitioners, and potential health risks—including the transmission of hepatitis or bacterial infections when procedures are performed by unlicensed individuals. The results of this study demonstrated that adolescents’ knowledge concerning appropriate piercing conditions, sterilization procedures, and serious complications was remarkably limited [5]. These findings highlight the need for educational interventions and health professional guidance on safe body modification practices. The EAP highlights a critical knowledge gap regarding viral transmission (e.g., HBV, HIV) and allergic reactions, which is directly reflected in our cohort’s moderate knowledge scores and their heavy reliance on non-medical information sources [3]. Furthermore, our finding that only half of the participants utilized licensed studios echoes the EAP’s concerns regarding inconsistent regulatory frameworks.

Clinically, our results reinforce the EAP’s recommendation that pediatricians should utilize body piercings as “dialogue openers” during routine consultations [3]. By adopting a nonjudgmental, “body-positive” approach, healthcare providers can facilitate anticipatory guidance on procedural safety while simultaneously screening for underlying mental health concerns, such as NSSI or depressive symptoms. Ultimately, integrating these discussions into adolescent primary care is essential for transforming a common form of self-expression into an opportunity for comprehensive psychosocial assessment and preventive intervention.

The study’s strengths include the clinical setting, structured assessment of both behavioral and psychosocial variables, and the inclusion of a validated suicide risk screening tool (ASQ). However, certain limitations should be acknowledged. The cross-sectional design precludes causal inference, and the sample was drawn from a single tertiary care center, which may limit generalizability. Self-reported data on behaviors such as substance use may also be subject to reporting bias. Future multicenter and longitudinal studies are warranted to further explore the temporal relationship between body modification practices and mental health outcomes in adolescents. While the clinical setting provides high internal validity, it may limit the generalizability of the findings to non-clinical environments. In such settings, the propensity for general risk behaviors may be lower, potentially influencing the applicability of our results as the limitation of our study. Future studies with longitudinal designs and larger sample sizes are warranted to confirm these findings and to further explore the underlying mechanisms.

## Conclusion

Body piercing among adolescents, while increasingly normalized as a form of self-expression and esthetic preference, remains associated with a cluster of health-risk behaviors and indicators of psychosocial vulnerability. These findings emphasize that body modification should not be viewed solely as a symbol of deviance, but rather as a potential marker of emotional distress or unmet psychosocial needs in certain youth populations. Piercing status appears to be a significant and independent marker of multiple risk behaviors, even after adjustment for key sociodemographic factors. These findings highlight the importance of considering body modification practices within a broader behavioral and psychosocial context.

Healthcare professionals—particularly pediatricians and adolescent health providers—should adopt a nonjudgmental, developmentally informed approach to discussing body modification, integrating education on safe piercing practices and screening for coexisting mental health and behavioral concerns. Future research should employ longitudinal designs to clarify causal pathways and to identify protective factors that can transform self-expression into a positive and safe developmental experience for adolescents.

## Supplementary Information

Below is the link to the electronic supplementary material.

ESM 1 (DOCX 14.6 KB)

## Data Availability

No datasets were generated or analysed during the current study.
