# Supplementary material for: Body piercing and adolescent risk-taking: association or expression?
Source: Eur J Pediatr. 2026 Jun 3;185(6):459. doi: 10.1007/s00431-026-07115-x (PMC13234038; doi:10.1007/s00431-026-07115-x)
Supplement: Supplementary file 1 — (DOCX 14.6 KB) [file 431_2026_7115_MOESM1_ESM.docx]

**SUPPLEMENTARY FILE**

Instructions: Please answer the following questions regarding your lifetime experiences. All responses are confidential.

1. Online-to-Offline Interaction: Have you ever met in person with someone you first encountered on the internet? (Yes/No)
2. Safety Negligence: Have you ever driven or traveled in a motor vehicle without wearing a seat belt? (Yes/No)
3. Physical Risk: Have you ever entered or traveled in a vehicle owned/driven by a stranger? (Yes/No)
4. Substance Use:

- Have you ever experimented with or used cigarettes? (Yes/No)
- Have you ever experimented with or used alcohol? (Yes/No)
- Have you ever experimented with or used illicit substances or drugs? (Yes/No)

1. Non-Suicidal Self-Injury (NSSI): Have you ever intentionally caused direct damage to your body tissue (e.g., cutting, burning) without the intent to die? (Yes/No)
